# Microproteomic-Based Analysis of the Goat Milk Protein Synthesis Network and Casein Production Evaluation

**DOI:** 10.3390/foods13040619

**Published:** 2024-02-19

**Authors:** Li Chen, Hiroaki Taniguchi, Emilia Bagnicka

**Affiliations:** 1 Department of Biotechnology and Nutrigenomics, Institute of Genetics and Animal Biotechnology, Polish Academy of Sciences, 05-552 Jastrzębiec, Poland; 2 College of Food Engineering and Nutritional Science, Shaanxi Normal University, Xi’an 710119, China; 3 Department of Experimental Embryology, Institute of Genetics and Animal Biotechnology, Polish Academy of Sciences, 05-552 Jastrzębiec, Poland; h.taniguchi@igbzpan.pl; 4 African Genome Center, University Mohammed VI Polytechnic (UM6P), Lot 660, Hay Moulay Rachid, Ben Guerir 43150, Morocco

**Keywords:** goat milk protein synthesis network, microproteomic analysis, *IRS1* gene, milk protein content evaluation

## Abstract

Goat milk has been consumed by humans since ancient times and is highly nutritious. Its quality is mainly determined by its casein content. Milk protein synthesis is controlled by a complex network with many signal pathways. Therefore, the aim of our study is to clearly depict the signal pathways involved in milk protein synthesis in goat mammary epithelial cells (GMECs) using state-of-the-art microproteomic techniques and to identify the key genes involved in the signal pathway. The microproteomic analysis identified more than 2253 proteins, with 323 pathways annotated from the identified proteins. Knockdown of *IRS1* expression significantly influenced goat casein composition (α, β, and κ); therefore, this study also examined the insulin receptor substrate 1 (IRS1) gene more closely. A total of 12 differential expression proteins (DEPs) were characterized as upregulated or downregulated in the *IRS1*-silenced sample compared to the negative control. The enrichment and signal pathways of these DEPs in GMECs were identified using GO annotation and KEGG, as well as KOG analysis. Our findings expand our understanding of the functional genes involved in milk protein synthesis in goats, paving the way for new approaches for modifying casein content for the dairy goat industry and milk product development.

## 1. Introduction

Goat milk has greater digestibility and alkalinity, as well as a higher buffering capacity, than cow’s milk. Therefore, it is highly praised for its unique nutritional and functional properties. It also has better emulsifying and foaming properties and is favored by manufacturers in developing new food products. Goat milk proteins also contain higher levels of certain amino acids, such as tryptophan and cysteine, compared to cow milk proteins and are believed to possess immunomodulatory, allergy management, anti-inflammatory, and antioxidant effects, as well as antimicrobial and anticancer properties [1,2]. Furthermore, people who are allergic to cow milk may feel comfortable with goat milk because of its lower lactose content and the different forms of proteins found therein [3,4,5,6].

Initial information on milk secretion was obtained from goats’ milk, and this has provided an insight into the processes occurring in mammary glands and cows’ udders. Milk protein is secreted by mammary epithelial cells (MECs), in which milk quality is strongly influenced by casein production [7]. Milk protein, consisting of approximately 80% casein and 20% whey, plays an important role in the production of cheese and other dairy products. Promoting milk production is a priority for food science in general, and the dairy goat sector is particularly in need of a way to increase casein content to ensure its development.

Due to the high kinase activity of insulin receptors, the mammary gland remains highly sensitive to insulin throughout pregnancy and the lactation period [8]. Milk protein synthesis requires the activity of insulin, amino acids, and amino acid transporters, as well as the mammalian target of rapamycin (mTOR) pathway [9,10,11].

To better understand the pathways of milk protein synthesis, proteomic techniques have been used to investigate the functional proteins in animal tissues [12,13,14]. Although standard (macro)proteomic application is suitable for large samples with protein losses measured in micrograms or milligrams, it is not sensitive enough for small numbers of cell samples. Moreover, sample preparation consists of several steps that can lead to protein loss, thus reducing the levels of low-abundance proteins and preventing their accurate identification. Fortunately, microproteomic (μP) approaches have been developed for the analysis of samples with attomolar protein concentrations, where even proteins present in sub-microgram levels can be analyzed while retrieving useful proteome data [15,16].

To date, no μP pipeline analyses have been performed on milk protein synthesis pathways in goat mammary epithelial cells (GMECs). Therefore, the present study examines the pathways of milk protein synthesis in GMECs using μP pipelines with the aid of state-of-the-art mass spectrometers and Orbitrap instruments. The results will shed greater light on the key genes taking part in milk protein synthesis networks and provide a novel insight into milk protein synthesis mechanisms in GMECs.

## 2. Materials and Methods

### 2.1. Cell Culture

The purified primary GMECs were donated by Prof. Jernej Ogorevc from the University of Ljubljana, Slovenia. Mammary tissue was obtained from slaughtered lactating Saanen goats (*Capra hircus*), which were approximately three years old at the peak of lactation [17]. The purified fourth-passage cells were selected with the basal medium, including 90% DMEM/F12 (11320033, Gibco, Thermo Fisher Scientific, Waltham, MA, USA), 10% fetal bovine serum (E5050, EURX, Gdańsk, Poland), 1% penicillin–streptomycin supplemented with 1 µg/mL of hydrocortisone (H6909, BioXtra, Sigma-Aldrich, Darmstadt, Germany), 10 ng/mL of epidermal growth factor (PHG0311, Gibco, Thermo Fisher Scientific, Waltham, MA, USA), 5 µg/mL of insulin solution from bovine pancreas (I0516, BioReagent, Sigma-Aldrich, Darmstadt, Germany), and L-glutamine (G7513, BioXtra, Sigma-Aldrich, Darmstadt, Germany) at a final concentration of 4.5 mM [17].

### 2.2. Microproteomic Analysis

A high-resolution mass spectrometer (MS) was used to analyze the microsample data. The microsample data of MS were processed with MaxQuant’s integrated Andromeda engine and the “match between runs” mode [15]. The analysis was based on peptide peak intensity, peak area, and LC retention time related to MS1, as well as other information. The data were subjected to statistical analysis and quality control before the GO, KOG, pathway, and other functional annotation analyses.

#### 2.2.1. Microsample Protein Extraction and Enzymolysis

Protein extraction and enzymolysis were performed by BGI Genomics Co., Ltd. (Shenzhen, China). The cell sample was mixed with 10 μL 50 mM ammonium bicarbonate, subjected to ultrasonic lysis for 10 min, and incubated with DL-Dithiothreitol (DTT) at a final concentration of 10 mM in a water bath at 37 °C for 30 min. Following this, iodoacetamide solution (IAM) was added to a final concentration of 55 mM and left to react for 45 min in the dark. Finally, 1 μg trypsin was added for enzymatic hydrolysis at 37 °C for two hours [15].

#### 2.2.2. Microsample MS Analysis

Protein separation was performed using a Thermo UltiMate 3000 UHPLC through a trap column and a self-packed C18 column at a flow rate of 500 nL/min. Peptide separation for DDA (data-dependent acquisition) mode was performed using a combined nanoESI source and Orbitrap Fusion™ Lumos™ Tribrid™ (Thermo Fisher Scientific, San Jose, CA, USA). The identification data were selected at PSM-level FDR ≤ 1%, and the significant identification was collected at protein-level FDR ≤ 1% [15].

### 2.3. Differential Quantification Analysis

The proteins identified in each sample were quantified using MaxQuant to determine their levels in each sample [18]. The data were subjected to Welch’s *t*-test to test the preset comparison group and calculate the multiple of differences. Significant differences were indicated by a fold change > 1.5 and *p* value < 0.05.

### 2.4. Bioinformatics Analysis

In all samples, proteins were identified using Gene Ontology (GO) functional annotation analysis [19]. The GO analysis was based on three ontologies (cellular component, biological process, and molecular function); the IDs and the number of proteins of all the corresponding proteins were listed. The identified proteins were classified into functional divisions using eukaryotic orthologous group (KOG) annotation according to the KOG database. The Kyoto Encyclopedia of Genes and Genomes (KEGG) pathway database was used to help further understand their biological functions.

### 2.5. RNAi

The siRNA used in this study was synthesized by Merck Life Science (Poznań, Poland) (Table 1). RNAi was performed by Lipofectamine™ RNAiMAX Transfection Reagent (13778075, Invitrogen, Thermo Fisher, Waltham, MA, USA) with the Opti-MEM^®^ I Reduced Serum Medium (31985070, Gibco, Thermo Fisher, Waltham, MA, USA) [20]. MISSION^®^ siRNA Universal Negative Control #1 (SIC001, Sigma-Aldrich, Darmstadt, Germany) was used as a negative control at a concentration of 20 μM. The cells were combined with a transfection mixture at a concentration of 0.15 × 10^6^/mL and then incubated for 48 h at 37 °C and 5% CO_2_. RNA was isolated to determine silencing efficiency.

### 2.6. RNA Isolation and Reverse Transcription Quantitative PCR (RT-qPCR)

Total RNA was isolated and purified with a NucleoSpin RNA Mini kit for RNA purification (Macherey-Nagel GmbH & Co. KG, Düren, Germany). RNA quantity and purity were determined using a Nanodrop 1000 (Thermo Scientific, Waltham, MA, USA) and a Bioanalyzer (Agilent 2100, Santa Clara, CA, USA). RNA was reverse transcribed with a Transcriptor First Strand cDNA synthesis kit (Roche, LifeScience Solutions, Basel, Switzerland) with random hexamer primers, according to the manufacturer’s instructions [20]. The final concentration of total RNA was approximately 595 ng/µL in all samples for cDNA synthesis. The relative expression of genes was determined by RT-qPCR. Glyceraldehyde-3-phosphate dehydrogenase (GAPDH) was adopted as a reference. The primers were copied from previous studies on goat cells (Table 2).

Expression analysis was performed using a LightCycler 480 SYBR Green I Master (Roche, Basel, Switzerland) using at least three technical replicates for each sample [20]. The amplification reactions contained 2× Master Mix, 10× each PCR primer (0.4 µM), and water, to a total volume of 20 µL. The following sequence was performed: pre-incubation at 95 °C for 10 min, followed by amplification for 45 cycles at 95 °C for 10 s, 60 °C for 10 s, and 72 °C for 10 s. The melting curve was 95 °C for 5 s and 65 °C and 97 °C for 1 min. The 2^−ΔΔCt^ method was adopted to calculate relative gene expression.

### 2.7. Milk Protein Secretion Determination

The protein content was determined by the goat casein alpha (CSN1) ELISA kit, goat beta-casein (Csn2) ELISA kit, and goat kappa casein (κ-CN) ELISA kit. The absorbance was measured at OD450 nm with a microplate reader (TECAN F039300, Männedorf, Switzerland). All calculations were performed using CurveExpert Professional 2.6.5 software. All reagents were obtained from the Wuhan Xinqidi Biological technology Co., Ltd. (Wuhan, China).

### 2.8. Statistical Analysis

All the experiments were repeated three times with three replicates. All results were analyzed using Duncan’s multiple-range tests (*p* < 0.05) by SAS 9.0 software (Cary, NC, USA).

## 3. Results

### 3.1. Pathway Annotation Analysis of GMEC Proteins

Protein function is associated with its biological behavior, which is related to many complex signal transduction pathways. The most important biochemical metabolic pathways and the signal transduction pathways formed by a protein can be determined by pathway analysis. Our present findings indicate the presence of more than 2253 proteins and about 337 pathway annotations among the quantified key proteins in GMECs (Table S1). About 42 of the identified proteins have been recorded in the insulin signaling pathway, and 44 in the mTOR signaling pathway. Milk protein synthesis is known to involve many complex factors [22]. The insulin–mTOR signal pathway merited particular attention because insulin has been reported to directly stimulate mTOR protein activity through phosphorylation [23].

### 3.2. Relative Quantitation of IRS1 Expression during Silence

The proteins belonging to the Insulin Receptor Substrate (IRS) family, IRS1, IRS2, IRS3, and IRS4, play a vital role in insulin signal transduction [24,25]. All four are phosphorylated on multiple tyrosine residues following insulin receptor kinase activation [26]. Previous studies found IRS1 to remarkably affect insulin-like growth factor and stimulate growth [27]. IRS1-deficient mice have mild glucose intolerance and insulin resistance [28]. IRS1 has also been found to be downregulated and to play a key role in cell proliferation and survival in breast cancer [29,30].

**Figure 1:**
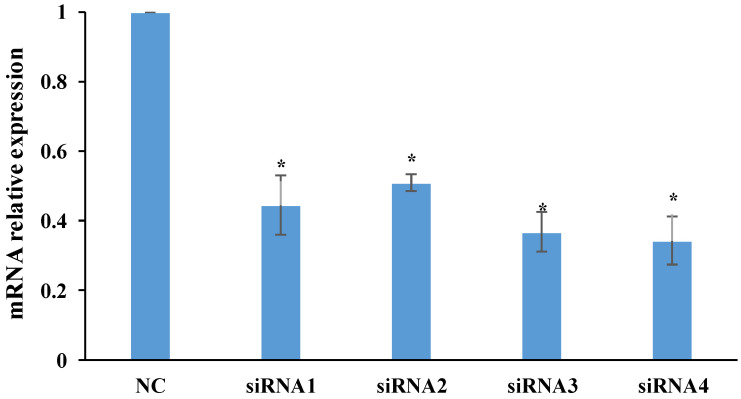
RT-qPCR analysis of *IRS1* expression in silenced GMECs. Relative gene expression was determined after transfection with negative control (NC), Lipo (lipofectamine™ RNAiMAX), siRNA1, siRNA2, siRNA3, and siRNA4 in GMEC for 48 h. The results are shown as mean ± SD, and the statistically significant analysis was calculated by Duncan’s multiple-range tests. The asterisks indicate statistically significant differences, *p* < 0.05.

The present study used four pairs of synthetic siRNAs to silence the *IRS1* gene and then measure the relative expression of mRNA in all samples using RT-qPCR. It was found that mRNA expression was significantly reduced in all four siRNA samples compared to the negative control (NC), indicating successful blockage by the four synthetic siRNAs (Figure 1). The samples treated with the four siRNAs demonstrated similar mRNA expression, with no statistically significant difference between them.

### 3.3. Casein Production Detection of GMECs

**Figure 2:**
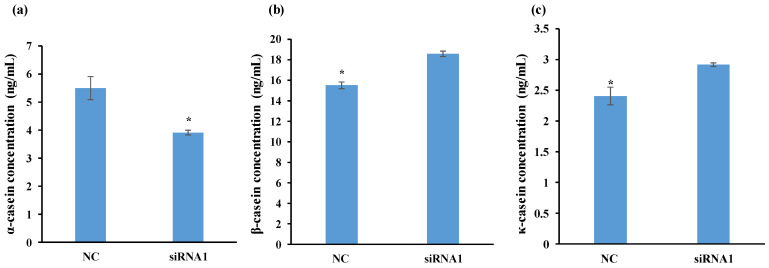
Determination of the content of (**a**) αs-1, (**b**) β-casein, and (**c**) κ-casein in GMECs. The casein content was determined after transfection with negative control (NC), Lipo (lipofectamine™ RNAiMAX), and siRNA1 in GMEC for 48 h. The column represents the mean ± SD; statistically significant differences were calculated by Duncan’s multiple-range tests; the asterisks indicate statistically significant differences, *p* < 0.05.

Goat milk protein consists of approximately 80% casein and 20% whey. The two have unique properties that can support the conversion of milk into yogurt and cheese. In turn, goat milk casein consists of four principal proteins: α_s1_-casein (α_s1_-CN), α_s2_-casein (α_s2_-CN), β-casein (β-CN), and κ-casein (κ-CN) [1]. Of these, β-casein is the most abundant in goat milk, and the allergen α_s1_-casein is the most abundant in cow milk. As shown in Figure 2, the content of κ-, β-, and α-casein differed significantly between *IRS1*-silenced cells and controls: κ- and β-casein contents were higher, while α-casein content was lower. As the samples treated with the four siRNAs demonstrated similar casein contents, siRNA1 was selected for further study.

### 3.4. Identification of Differential Expression Proteins (DEPs) by Microproteomic Analysis

**Figure 3:**
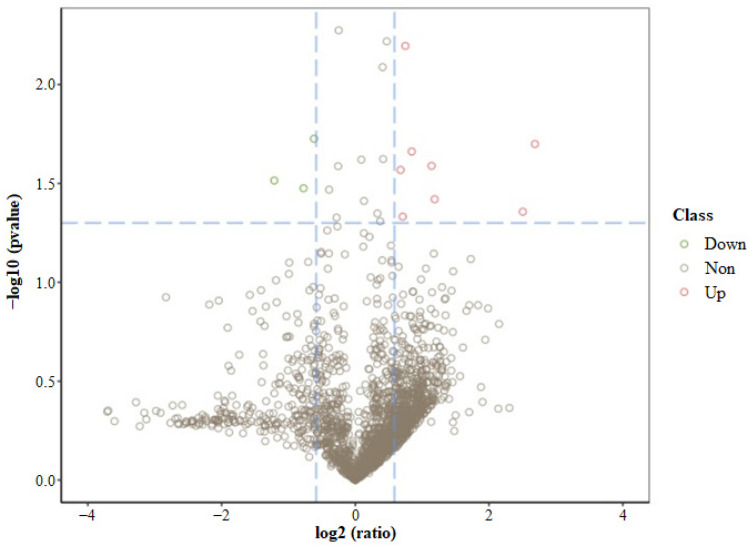
Volcano plot of screened DEPs. The *x*-axis indicates the protein difference multiple, while the *y*-axis is the −log10 (*p* value). A gray dot indicates a non-significantly altered protein (following silencing), a red dot indicates an upregulated protein, and a green dot indicates a downregulated protein.

The DEPs in the test samples are depicted in volcano plots in Figure 3. Nine DEPs in the siRNA1 sample were found to be upregulated, and three were downregulated compared to the NC samples in GMECs (Table S2). The upregulated DEPs were identified as Keratin, MAP7 domain, Syntaxin, KIAA1217 ortholog, Phosphodiesterase, Heme binding protein, Rhophilin Rho GTPase binding protein, and Myosin XVIIIA. The downregulated proteins were Protein arginine N-methyltransferase, Glutaredoxin, and Protein MAK16.

### 3.5. KOG Analysis of the DEPs in GMECs

**Figure 4:**
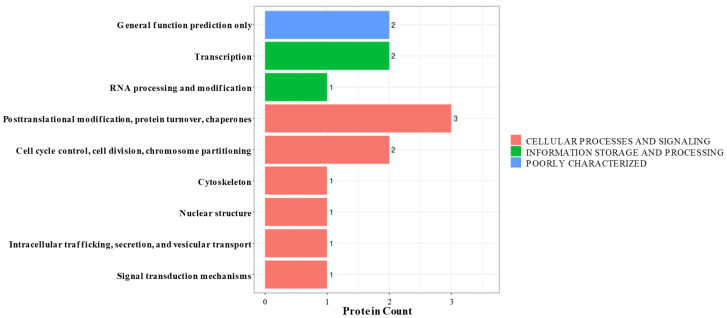
DEPs’ KOG annotation. The *x*-axis represents protein count, and the *y*-axis represents KOG terms.

KOG analysis was used to classify DEPs in the NC vs. siRNA1 samples into three divisions: cellular process and signaling, information storage and processing, and poorly characterized. In Figure 4, it can be seen that most DEPs belong to the cellular process and signaling division: post-translational modification, protein turnover, and chaperones. Others were classified as information storage and processing, with the most common function being transcription. Finally, in the poorly characterized division, general function prediction only was noted.

### 3.6. GO Analysis of DEPs

**Figure 5:**
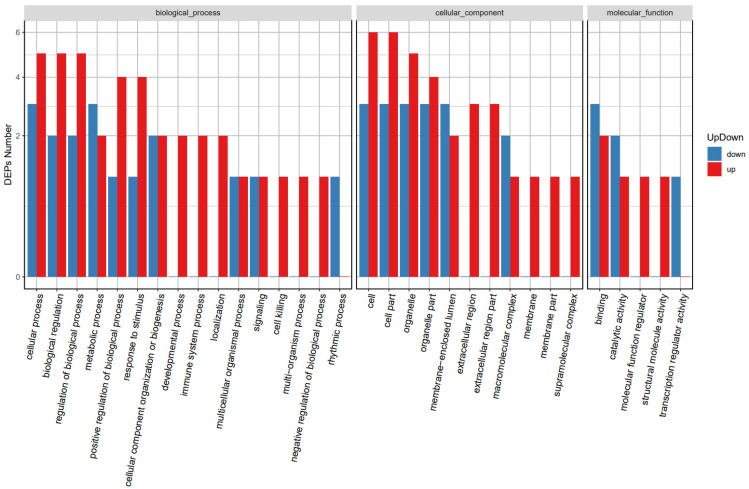
DEP GO function classification up and down chart. The *x*-axis is GO annotation, and the *y*-axis is DEP number.

The DEPs in GMECs in NC vs. siRNA1 were classified into the cellular composition, biological processes, and molecular function groups according to GO annotation (Table S3). The GO function up and down chart of the DEPs is given in Figure 5. In the *biological process* division, the most upregulated proteins belong to biological regulation, cellular process, and regulation of biological processes, while the major downregulated proteins belong to cellular process and metabolic process. In the *cellular component* division, both the most upregulated and downregulated proteins belonged to the cell, cell part, and organelle groups. Finally, in *molecular function*, the most upregulated components belonged to binding, while the most downregulated proteins belonged to binding and catalytic activity.

**Figure 6:**
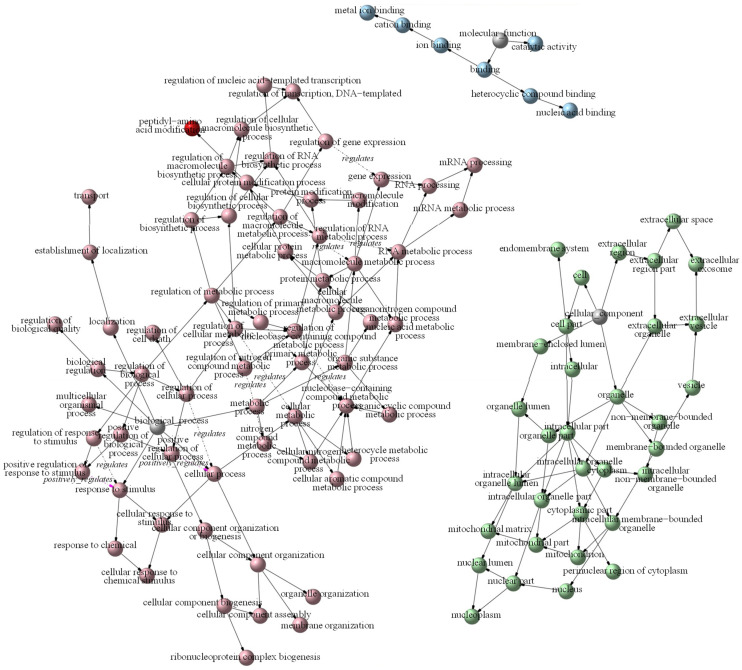
GO term relationship network diagram. A node is a GO term. The colors indicate different functional categories. Red indicates biological processes, green cellular components, and blue molecular functions. Dark colors indicate significantly enriched GO terms, light colors indicate insignificant GO terms, and grays indicate no enriched GO terms. A solid arrow indicates an inclusion relationship between GO terms, while a dotted arrow indicates a regulation relationship. A red dotted line suggests positive regulation, and a green dotted line negative regulation.

A GO term relationship network was established to describe DEP enrichment (Figure 6). In the diagram, a node indicates a GO term. Green indicates cellular components, red biological processes, and blue molecular functions. *Biological processes* included two positive regulations (cellular process and response to stimulus) and nine negative regulations (RNA metabolic process, cellular metabolic process, macromolecule metabolic process, and nucleobase-containing compound metabolic process). No GO term regulation was observed in the *cellular component* or *molecular function* divisions.

### 3.7. KEGG Pathway Analysis of DEPs

**Figure 7:**
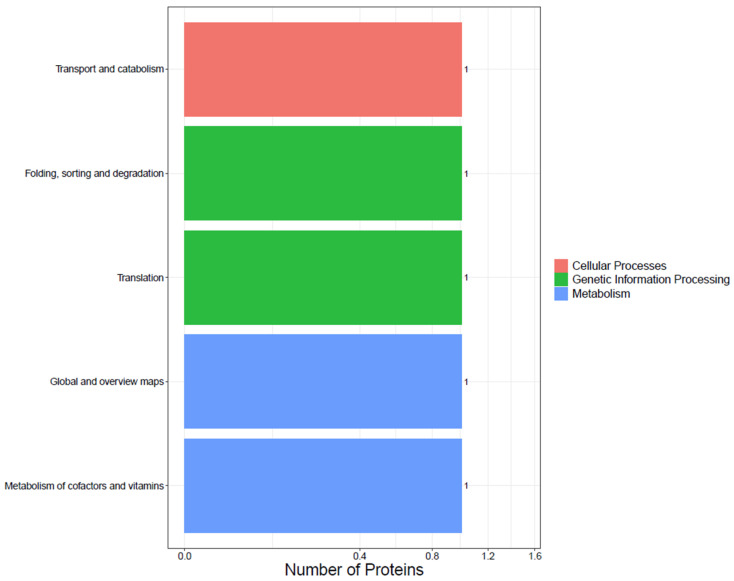
DEP pathway classification by KEGG enrichment. The *x*-axis represents protein number, and the *y*-axis represents KEGG pathway enrichment.

The KEGG pathway analyses classified the DEPs into cellular process, genetic information processing, and metabolism divisions (Figure 7). The main pathways involved were folding, sorting and degradation, translation, global and overview maps, and metabolism of cofactors and vitamins.

### 3.8. Subcellular Localization Prediction of DEPs

**Figure 8:**
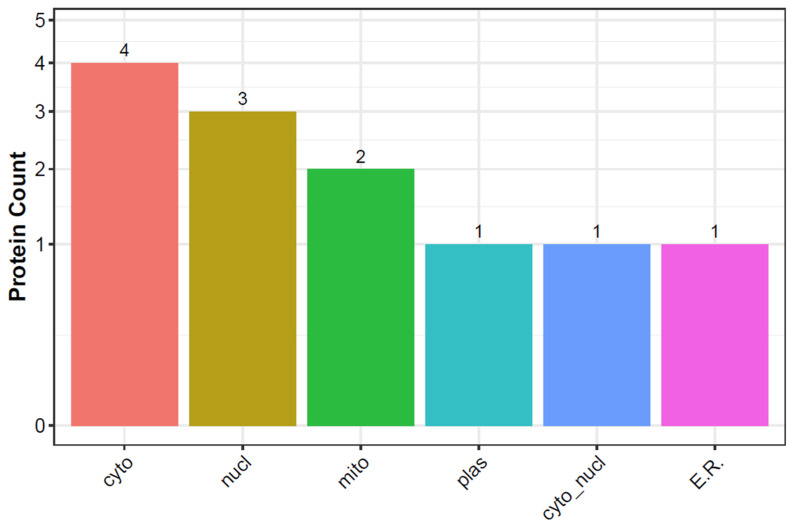
Subcellular localization prediction. The *x*-axis represents subcellular structure, and the *y*-axis represents protein number.

Subcellular localization prediction refers to the computational task of determining the specific location of a protein within a cell. Proteins perform their functions within specific compartments or organelles within the cell. Understanding the subcellular localization of molecules and organelles is essential for studying cellular processes, signaling pathways, and the mechanisms underlying health and disease. Subcellular localization prediction can be crucial for understanding the functions of DEPs within cells, as it can provide insights into their functions and roles within cellular processes. The DEPs were classified into six divisions: plasma membrane (plas), cytosol (cyto), mitochondrion (mito), nucleus (nucl), cytosol and nucleus (cyto_nucl), and endoplasmic reticulum (E.R.) (Table S4). The main subcellular locations of DEPs were cyto, nucl, and mito (Figure 8).

## 4. Discussion

Milk protein is secreted by mammary epithelial cells (MECs), and casein content is a key determinant of milk quality [31]. Recent years have seen a number of studies aimed at increasing milk protein secretion in MECs based on molecular mechanisms and signal pathways [32,33]. However, milk protein synthesis is a complex process. AMP-activated protein kinase (AMPK) and tumor suppressor LKB1 are located upstream of mTOR [34]. AMPK activates ATP-generating pathways and inhibits ATP consumption. The inhibition of mTORC1 mediated by LKB1 relies on AMPK and TSC2. Milk protein synthesis also involves the insulin–mTOR pathway. All these signaling pathways have been confirmed by our μP approaches.

As shown in Table S1, about 66 of the proteins identified in GMECs belong to the PI3K-Akt signaling pathway, which has been shown to play an important role in camel milk protein networks [35]. Interference in the PI3K-Akt signal may lead to insulin resistance, resulting in the creation of a vicious circle [36]. Additionally, many of the identified proteins were found to be associated with more than 330 pathways, including MAPK signaling, insulin signaling, necroptosis, apoptosis, biosynthesis of amino acids, AMPK signaling, mTOR signaling, and TNF signaling; some of these are closely linked with milk protein synthesis (Table S1). Previous studies indicate that the insulin–mTOR pathway plays a role in regulating milk protein synthesis, with insulin directly stimulating the mTOR protein.

The IRS-family proteins are closely associated with the insulin signal pathway [37,38]. Indeed, IRS1 can be found in the central part of a signaling pathway network diagram of camel milk proteins designed by Han (2022) [35]. Our study explored the role of *IRS1* in goat milk synthesis and casein composition. Casein plays an important role in cheese making as it dictates how well, and how rapidly, the milk clots and forms a curd. Any changes in the amount of α-CN or β-CN would alter the properties of the milk and the resulting cheese [39]. Our findings indicate that *IRS1* silencing significantly influenced the content of κ-casein, β-casein, and α-casein in GMECs (Figure 2).

Previous studies found goat milk with altered α_S1_-CN contents to be allergenic in guinea pigs [40]. Goat milk lacking α_S1_-CN was less allergenic than other goat milks, probably due to its modified ratio of β-LG to α_S_-CN. In the present study, the *IRS1*-silenced sample demonstrated higher levels of β-casein and lower levels of α-casein. Unfortunately, as little is currently known about casein synthesis, it is hard to explain these changes. Nevertheless, these findings encourage further research in the area.

In the present study, microproteomic analysis identified about 12 DEPs among more than 2253 proteins (Figure 3 and Table S2). Among these, the upregulated DEPs were Keratin, MAP7 domain, Syntaxin, KI-AA1217 ortholog, Phosphodiesterase, Heme binding protein, Rhophilin Rho GTPase binding protein, and Myosin XVIIIA. The downregulated DEPs were Protein arginine N-methyltransferase, Glutaredoxin, and Protein MAK16.

Syntaxin is involved in vesicle trafficking and membrane fusion events within cells, particularly during exocytosis [41], the cellular process in which substances are released from vesicles into the extracellular space. Although syntaxin itself is not directly implicated in the synthesis of milk proteins, proteins involved in vesicle trafficking, membrane fusion, and intracellular transport could indirectly impact the secretion of milk proteins. These processes are crucial for the proper packaging and release of proteins from cells, including the MECs responsible for milk production.

Phosphodiesterases play a crucial role in intracellular signaling by hydrolyzing cyclic nucleotides, particularly cyclic guanosine monophosphate (cGMP) and cyclic adenosine monophosphate (cAMP) [42]. These cyclic nucleotides are involved in signaling pathways that regulate various cellular processes. Although there may not be a direct link between phosphodiesterase and milk protein synthesis, alterations in cyclic nucleotide levels regulated by phosphodiesterases could potentially influence cellular processes and signaling pathways, indirectly impacting milk protein synthesis. Protein Arginine N-Methyltransferase belongs to a family of enzymes involved in the methylation of arginine residues in proteins. They play various roles in cellular processes, including gene expression regulation, signal transduction, and RNA processing [43].

All these upregulated and downregulated proteins were associated with the modified casein composition in GMECs. Doubtlessly, changes in the levels of α-CN or β-CN would alter the properties of milk and the produced cheese, influencing their processing. Furthermore, increasing evidence indicates that β-asomorphin-7 derived from A1 β-casein contributes to milk intolerance syndrome. Our findings provide interesting information for the fields of milk processing and nutrition mechanisms.

The GO annotations found two DEPs to be upregulated, with these associated with cellular process and response to stimulus, and nine to be downregulated, related to RNA metabolic process, cellular metabolic process, macromolecule metabolic process, and nucleobase-containing compound metabolic process (Figure 6).

Milk protein synthesis is a complex process that occurs in the mammary glands and is generally associated with hormonal signals, nutritional factors, and the specific needs of the developing offspring [32,44]. Therefore, to better understand the role of IRS1 in goat mammary glands and milk protein synthesis, further functional studies of the proteins influenced by IRS1 silencing in GMECs are needed.

## 5. Conclusions

Our findings confirm that the *IRS1* gene influences the casein content of milk in goats and the milk protein synthesis pathways in GMECs. Modifying the expression of *IRS1* could increase the amount of κ-casein and β-casein but decrease the content of α-casein. This study is the first to successfully use a microproteomic approach to analyze the proteins of GMECs with low amount requirements. By identifying the proteins that were differentially expressed in response to *IRS1* silencing, it was possible to gain a new insight into the goat milk protein synthesis network and related signal pathways. Some DEPs were found to indirectly influence milk protein synthesis based on their GO annotation and their KEGG and KOG analysis. These findings may have positive implications for future studies on the milk synthesis system in goats.

## Figures and Tables

**Table 1:** The design of siRNA.

Target Name	Sense/Antisense	siRNA Design	Start on Target	Target Sequence
IRS1	s	CUACCAUUUCCCACCAGAAdTdT	1595	CTACCATTTCCCACCAGAA
IRS1	a	UUCUGGUGGGAAAUGGUAGdTdT	1595	TTCTGGTGGGAAATGGTAG
IRS1	s	CACUUUACCUCGGGCCCGAdTdT	2607	CACTTTACCTCGGGCCCGA
IRS1	a	UCGGGCCCGAGGUAAAGUGdTdT	2607	TCGGGCCCGAGGTAAAGTG
IRS1	s	CAUUGAGGAAUAUACUGAAdTdT	1641	CATTGAGGAATATACTGAA
IRS1	a	UUCAGUAUAUUCCUCAAUGdTdT	1641	TTCAGTATATTCCTCAATG
IRS1	s	CAAAGAACCUGAUUGGCAUdTdT	527	CAAAGAACCTGATTGGCAT
IRS1	a	AUGCCAAUCAGGUUCUUUGdTdT	527	ATGCCAATCAGGTTCTTTG

**Table 2:** Primers for RT-qPCRs.

Gene	Sequence	Reference
GAPDH	For 5′-CATGTTTGTGATGGGCGTGAACCA-3′, Rev 5′-TAAGTCCCTCCACGATGCCAAAGT-3	[17]
IRS1	IRS1-F GTAGTGGCAAACTCCTGTCTTGT, IRS1-R GAGTAGTAGGAGAGGACGGGCT	[21]

## Data Availability

The original contributions presented in the study are included in the article/supplementary material, further inquiries can be directed to the corresponding authors.

## Supplementary Materials

The following supporting information can be downloaded at https://www.mdpi.com/article/10.3390/foods13040619/s1: Table S1: Pathway annotation analysis of identified proteins in GMECs; Table S2: Identified DEPs in *IRS1*-silenced GMECs; Table S3: GO enriched DEPs; Table S4: Subcellular localization of DEPs.
