# Supplementary material for: Microproteomic-Based Analysis of the Goat Milk Protein Synthesis Network and Casein Production Evaluation
Source: Foods. 2024 Feb 19;13(4):619. doi: 10.3390/foods13040619 (PMC10887518; doi:10.3390/foods13040619)
Supplement: Supplementary file 1 [file foods-13-00619-s001.zip › foods-2861783-supplementary.pdf]

Supporting Information

Microproteomic-Based Analysis of the Goat Milk Protein Synthesis Network and Casein Production Evaluation

Li Chen [1,2,*], Hiroaki Taniguchi [3,4] and Emilia Bagnicka [1,*]

[1] Department of Biotechnology and Nutrigenomics, Institute of Genetics and Animal Biotechnology, Polish Academy of Sciences, 05-552 Jastrzębiec, Poland

[2] College of Food Engineering and Nutritional Science, Shaanxi Normal University, Xi'an, 710119, China

[3] Department of Experimental Embryology, Institute of Genetics and Animal Biotechnology, Polish Academy of Sciences, 05-552 Jastrzębiec, Poland; h.taniguchi@igbzpan.pl

[4] African Genome Center, University Mohammed VI Polytechnic (UM6P), Lot 660, Hay Moulay Rachid, Ben Guerir 43150, Morocco

* Correspondence: l.chen@igbzpan.pl (L.C.); e.bagnicka@igbzpan.pl (E.B.);
Tel.: +48-(22)-7367000 (E.B.)

Table S1. Pathway annotation analysis of identified proteins in GMECs

	Pathway	Proteins with pathway annotation (3055)	Pathway ID	Level1	Level2
**1**	Metabolic pathways	472 (15.45%)	ko01100	Metabolism	Global and overview maps
**2**	Pathways in cancer	121 (3.96%)	ko05200	Human Diseases	Cancers: Overview
**3**	Endocytosis	117 (3.83%)	ko04144	Cellular Processes	Transport and catabolism
**4**	Ribosome	111 (3.63%)	ko03010	Genetic Information Processing	Translation
**5**	Spliceosome	108 (3.54%)	ko03040	Genetic Information Processing	Transcription
**6**	Thermogenesis	108 (3.54%)	ko04714	Organismal Systems	Environmental adaptation
**7**	Human papillomavirus infection	106 (3.47%)	ko05165	Human Diseases	Infectious diseases: Viral
**8**	Huntington disease	103 (3.37%)	ko05016	Human Diseases	Neurodegenerative diseases
**9**	RNA transport	102 (3.34%)	ko03013	Genetic Information Processing	Translation
**10**	Alzheimer disease	100 (3.27%)	ko05010	Human Diseases	Neurodegenerative diseases
**11**	Regulation of actin cytoskeleton	95 (3.11%)	ko04810	Cellular Processes	Cell motility
**12**	Protein processing in endoplasmic reticulum	95 (3.11%)	ko04141	Genetic Information Processing	Folding, sorting and degradation
**13**	Oxidative phosphorylation	89 (2.91%)	ko00190	Metabolism	Energy metabolism
**14**	Parkinson disease	89 (2.91%)	ko05012	Human Diseases	Neurodegenerative diseases
**15**	Focal adhesion	89 (2.91%)	ko04510	Cellular Processes	Cellular community - eukaryotes
**16**	PI3K-Akt signaling pathway	86 (2.82%)	ko04151	Environmental Information Processing	Signal transduction
**17**	Tight junction	81 (2.65%)	ko04530	Cellular Processes	Cellular community - eukaryotes
**18**	Carbon metabolism	81 (2.65%)	ko01200	Metabolism	Global and overview maps
**19**	Human immunodeficiency virus 1 infection	78 (2.55%)	ko05170	Human Diseases	Infectious diseases: Viral
**20**	Proteoglycans in cancer	75 (2.45%)	ko05205	Human Diseases	Cancers: Overview
**21**	Viral carcinogenesis	75 (2.45%)	ko05203	Human Diseases	Cancers: Overview
**22**	Phagosome	74 (2.42%)	ko04145	Cellular Processes	Transport and catabolism
**23**	Non-alcoholic fatty liver disease (NAFLD)	72 (2.36%)	ko04932	Human Diseases	Endocrine and metabolic diseases
**24**	Epstein-Barr virus infection	68 (2.23%)	ko05169	Human Diseases	Infectious diseases: Viral
**25**	Human cytomegalovirus infection	66 (2.16%)	ko05163	Human Diseases	Infectious diseases: Viral
**26**	MAPK signaling pathway	66 (2.16%)	ko04010	Environmental Information Processing	Signal transduction
**27**	Influenza A	62 (2.03%)	ko05164	Human Diseases	Infectious diseases: Viral
**28**	Cellular senescence	56 (1.83%)	ko04218	Cellular Processes	Cell growth and death
**29**	Necroptosis	56 (1.83%)	ko04217	Cellular Processes	Cell growth and death
**30**	Herpes simplex infection	55 (1.8%)	ko05168	Human Diseases	Infectious diseases: Viral
**31**	Apoptosis	54 (1.77%)	ko04210	Cellular Processes	Cell growth and death

**32**	Lysosome	53 (1.73%)	ko04142	Cellular Processes	Transport and catabolism
**33**	Human T-cell leukemia virus 1 infection	53 (1.73%)	ko05166	Human Diseases	Infectious diseases: Viral
**34**	Ras signaling pathway	52 (1.7%)	ko04014	Environmental Information Processing	Signal transduction
**35**	mRNA surveillance pathway	52 (1.7%)	ko03015	Genetic Information Processing	Translation
**36**	Hepatitis C	52 (1.7%)	ko05160	Human Diseases	Infectious diseases: Viral
**37**	Retrograde endocannabinoid signaling	51 (1.67%)	ko04723	Organismal Systems	Nervous system
**38**	Bacterial invasion of epithelial cells	51 (1.67%)	ko05100	Human Diseases	Infectious diseases: Bacterial
**39**	Kaposi sarcoma-associated herpesvirus infection	49 (1.6%)	ko05167	Human Diseases	Infectious diseases: Viral
**40**	Rap1 signaling pathway	49 (1.6%)	ko04015	Environmental Information Processing	Signal transduction
**41**	MicroRNAs in cancer	49 (1.6%)	ko05206	Human Diseases	Cancers: Overview
**42**	Biosynthesis of amino acids	49 (1.6%)	ko01230	Metabolism	Global and overview maps
**43**	Platelet activation	49 (1.6%)	ko04611	Organismal Systems	Immune system
**44**	Estrogen signaling pathway	48 (1.57%)	ko04915	Organismal Systems	Endocrine system
**45**	Adrenergic signaling in cardiomyocytes	47 (1.54%)	ko04261	Organismal Systems	Circulatory system
**46**	Hippo signaling pathway	46 (1.51%)	ko04390	Environmental Information Processing	Signal transduction
**47**	Ubiquitin mediated proteolysis	46 (1.51%)	ko04120	Genetic Information Processing	Folding, sorting and degradation
**48**	cGMP-PKG signaling pathway	46 (1.51%)	ko04022	Environmental Information Processing	Signal transduction
**49**	Tuberculosis	46 (1.51%)	ko05152	Human Diseases	Infectious diseases: Bacterial
**50**	Leukocyte transendothelial migration	46 (1.51%)	ko04670	Organismal Systems	Immune system
**51**	Salmonella infection	46 (1.51%)	ko05132	Human Diseases	Infectious diseases: Bacterial
**52**	Purine metabolism	45 (1.47%)	ko00230	Metabolism	Nucleotide metabolism
**53**	NOD-like receptor signaling pathway	45 (1.47%)	ko04621	Organismal Systems	Immune system
**54**	Fluid shear stress and atherosclerosis	44 (1.44%)	ko05418	Human Diseases	Cardiovascular diseases
**55**	Ribosome biogenesis in eukaryotes	44 (1.44%)	ko03008	Genetic Information Processing	Translation
**56**	mTOR signaling pathway	44 (1.44%)	ko04150	Environmental Information Processing	Signal transduction
**57**	Proteasome	44 (1.44%)	ko03050	Genetic Information Processing	Folding, sorting and degradation
**58**	Hepatocellular carcinoma	43 (1.41%)	ko05225	Human Diseases	Cancers: Specific types
**59**	Pathogenic Escherichia coli infection	43 (1.41%)	ko05130	Human Diseases	Infectious diseases: Bacterial
**60**	Axon guidance	43 (1.41%)	ko04360	Organismal Systems	Development
**61**	cAMP signaling pathway	43 (1.41%)	ko04024	Environmental Information Processing	Signal transduction
**62**	Oocyte meiosis	43 (1.41%)	ko04114	Cellular Processes	Cell growth and death
**63**	Chemokine signaling pathway	43 (1.41%)	ko04062	Organismal Systems	Immune system
**64**	Hepatitis B	42 (1.37%)	ko05161	Human Diseases	Infectious diseases: Viral
**65**	Insulin signaling pathway	42 (1.37%)	ko04910	Organismal Systems	Endocrine system
**66**	Oxytocin signaling pathway	41 (1.34%)	ko04921	Organismal Systems	Endocrine system
**67**	Autophagy - animal	40 (1.31%)	ko04140	Cellular Processes	Transport and catabolism

**68**	Dilated cardiomyopathy (DCM)	40 (1.31%)	ko05414	Human Diseases	Cardiovascular diseases
**69**	Sphingolipid signaling pathway	40 (1.31%)	ko04071	Environmental Information Processing	Signal transduction
**70**	Shigellosis	40 (1.31%)	ko05131	Human Diseases	Infectious diseases: Bacterial
**71**	Valine, leucine and isoleucine degradation	40 (1.31%)	ko00280	Metabolism	Amino acid metabolism
**72**	Glycolysis / Gluconeogenesis	38 (1.24%)	ko00010	Metabolism	Carbohydrate metabolism
**73**	Adherens junction	37 (1.21%)	ko04520	Cellular Processes	Cellular community - eukaryotes
**74**	RNA degradation	37 (1.21%)	ko03018	Genetic Information Processing	Folding, sorting and degradation
**75**	Cardiac muscle contraction	37 (1.21%)	ko04260	Organismal Systems	Circulatory system
**76**	Alcoholism	36 (1.18%)	ko05034	Human Diseases	Substance dependence
**77**	Complement and coagulation cascades	36 (1.18%)	ko04610	Organismal Systems	Immune system
**78**	Cell cycle	36 (1.18%)	ko04110	Cellular Processes	Cell growth and death
**79**	Toxoplasmosis	36 (1.18%)	ko05145	Human Diseases	Infectious diseases: Parasitic
**80**	Apelin signaling pathway	36 (1.18%)	ko04371	Environmental Information Processing	Signal transduction
**81**	Hypertrophic cardiomyopathy (HCM)	36 (1.18%)	ko05410	Human Diseases	Cardiovascular diseases
**82**	HIF-1 signaling pathway	35 (1.15%)	ko04066	Environmental Information Processing	Signal transduction
**83**	Fc gamma R-mediated phagocytosis	35 (1.15%)	ko04666	Organismal Systems	Immune system
**84**	Dopaminergic synapse	35 (1.15%)	ko04728	Organismal Systems	Nervous system
**85**	Neurotrophin signaling pathway	34 (1.11%)	ko04722	Organismal Systems	Nervous system
**86**	Amoebiasis	34 (1.11%)	ko05146	Human Diseases	Infectious diseases: Parasitic
**87**	Amino sugar and nucleotide sugar metabolism	33 (1.08%)	ko00520	Metabolism	Carbohydrate metabolism
**88**	ECM-receptor interaction	33 (1.08%)	ko04512	Environmental Information Processing	Signaling molecules and interaction
**89**	AGE-RAGE signaling pathway in diabetic complications	33 (1.08%)	ko04933	Human Diseases	Endocrine and metabolic diseases
**90**	Measles	32 (1.05%)	ko05162	Human Diseases	Infectious diseases: Viral
**91**	Vibrio cholerae infection	32 (1.05%)	ko05110	Human Diseases	Infectious diseases: Bacterial
**92**	MAPK signaling pathway - fly	32 (1.05%)	ko04013	Environmental Information Processing	Signal transduction
**93**	Legionellosis	32 (1.05%)	ko05134	Human Diseases	Infectious diseases: Bacterial
**94**	AMPK signaling pathway	32 (1.05%)	ko04152	Environmental Information Processing	Signal transduction
**95**	Fatty acid metabolism	31 (1.01%)	ko01212	Metabolism	Global and overview maps
**96**	Vascular smooth muscle contraction	31 (1.01%)	ko04270	Organismal Systems	Circulatory system
**97**	Relaxin signaling pathway	30 (0.98%)	ko04926	Organismal Systems	Endocrine system
**98**	Cushing syndrome	30 (0.98%)	ko04934	Human Diseases	Endocrine and metabolic diseases
**99**	Calcium signaling pathway	30 (0.98%)	ko04020	Environmental Information Processing	Signal transduction
**100**	Wnt signaling pathway	30 (0.98%)	ko04310	Environmental Information Processing	Signal transduction
**101**	Hippo signaling pathway - fly	30 (0.98%)	ko04391	Environmental Information Processing	Signal transduction
**102**	Drug metabolism - other enzymes	29 (0.95%)	ko00983	Metabolism	Xenobiotics biodegradation and metabolism

**103**	Pyruvate metabolism	29 (0.95%)	ko00620	Metabolism	Carbohydrate metabolism
**104**	Phospholipase D signaling pathway	29 (0.95%)	ko04072	Environmental Information Processing	Signal transduction
**105**	Aminoacyl-tRNA biosynthesis	29 (0.95%)	ko00970	Genetic Information Processing	Translation
**106**	Viral myocarditis	29 (0.95%)	ko05416	Human Diseases	Cardiovascular diseases
**107**	Fatty acid degradation	28 (0.92%)	ko00071	Metabolism	Lipid metabolism
**108**	Epithelial cell signaling in Helicobacter pylori infection	28 (0.92%)	ko05120	Human Diseases	Infectious diseases: Bacterial
**109**	Arrhythmogenic right ventricular cardiomyopathy (ARVC)	28 (0.92%)	ko05412	Human Diseases	Cardiovascular diseases
**110**	Gap junction	28 (0.92%)	ko04540	Cellular Processes	Cellular community - eukaryotes
**111**	Citrate cycle (TCA cycle)	28 (0.92%)	ko00020	Metabolism	Carbohydrate metabolism
**112**	Chagas disease (American trypanosomiasis)	28 (0.92%)	ko05142	Human Diseases	Infectious diseases: Parasitic
**113**	Cysteine and methionine metabolism	27 (0.88%)	ko00270	Metabolism	Amino acid metabolism
**114**	Renal cell carcinoma	27 (0.88%)	ko05211	Human Diseases	Cancers: Specific types
**115**	Thyroid hormone signaling pathway	27 (0.88%)	ko04919	Organismal Systems	Endocrine system
**116**	Synaptic vesicle cycle	27 (0.88%)	ko04721	Organismal Systems	Nervous system
**117**	IL-17 signaling pathway	27 (0.88%)	ko04657	Organismal Systems	Immune system
**118**	C-type lectin receptor signaling pathway	27 (0.88%)	ko04625	Organismal Systems	Immune system
**119**	Antigen processing and presentation	27 (0.88%)	ko04612	Organismal Systems	Immune system
**120**	Chronic myeloid leukemia	26 (0.85%)	ko05220	Human Diseases	Cancers: Specific types
**121**	Glucagon signaling pathway	26 (0.85%)	ko04922	Organismal Systems	Endocrine system
**122**	Glutathione metabolism	26 (0.85%)	ko00480	Metabolism	Metabolism of other amino acids
**123**	Platinum drug resistance	25 (0.82%)	ko01524	Human Diseases	Drug resistance: Antineoplastic
**124**	Arginine and proline metabolism	25 (0.82%)	ko00330	Metabolism	Amino acid metabolism
**125**	Gastric cancer	25 (0.82%)	ko05226	Human Diseases	Cancers: Specific types
**126**	Pertussis	25 (0.82%)	ko05133	Human Diseases	Infectious diseases: Bacterial
**127**	Colorectal cancer	25 (0.82%)	ko05210	Human Diseases	Cancers: Specific types
**128**	GnRH signaling pathway	25 (0.82%)	ko04912	Organismal Systems	Endocrine system
**129**	TNF signaling pathway	25 (0.82%)	ko04668	Environmental Information Processing	Signal transduction
**130**	Transcriptional misregulation in cancer	25 (0.82%)	ko05202	Human Diseases	Cancers: Overview
**131**	Central carbon metabolism in cancer	25 (0.82%)	ko05230	Human Diseases	Cancers: Overview
**132**	Cell adhesion molecules (CAMs)	25 (0.82%)	ko04514	Environmental Information Processing	Signaling molecules and interaction
**133**	Pyrimidine metabolism	24 (0.79%)	ko00240	Metabolism	Nucleotide metabolism
**134**	Small cell lung cancer	24 (0.79%)	ko05222	Human Diseases	Cancers: Specific types
**135**	Lysine degradation	24 (0.79%)	ko00310	Metabolism	Amino acid metabolism
**136**	Peroxisome	24 (0.79%)	ko04146	Cellular Processes	Transport and catabolism

**137**	FoxO signaling pathway	24 (0.79%)	ko04068	Environmental Information Processing	Signal transduction
**138**	T cell receptor signaling pathway	24 (0.79%)	ko04660	Organismal Systems	Immune system
**139**	Systemic lupus erythematosus	24 (0.79%)	ko05322	Human Diseases	Immune diseases
**140**	Toll-like receptor signaling pathway	23 (0.75%)	ko04620	Organismal Systems	Immune system
**141**	Long-term potentiation	23 (0.75%)	ko04720	Organismal Systems	Nervous system
**142**	NF-kappa B signaling pathway	23 (0.75%)	ko04064	Environmental Information Processing	Signal transduction
**143**	Insulin resistance	23 (0.75%)	ko04931	Human Diseases	Endocrine and metabolic diseases
**144**	Propanoate metabolism	23 (0.75%)	ko00640	Metabolism	Carbohydrate metabolism
**145**	Pancreatic cancer	23 (0.75%)	ko05212	Human Diseases	Cancers: Specific types
**146**	Progesterone-mediated oocyte maturation	23 (0.75%)	ko04914	Organismal Systems	Endocrine system
**147**	Longevity regulating pathway - worm	23 (0.75%)	ko04212	Organismal Systems	Aging
**148**	Ferroptosis	23 (0.75%)	ko04216	Cellular Processes	Cell growth and death
**149**	p53 signaling pathway	22 (0.72%)	ko04115	Cellular Processes	Cell growth and death
**150**	Osteoclast differentiation	22 (0.72%)	ko04380	Organismal Systems	Development
**151**	Glutamatergic synapse	22 (0.72%)	ko04724	Organismal Systems	Nervous system
**152**	ErbB signaling pathway	22 (0.72%)	ko04012	Environmental Information Processing	Signal transduction
**153**	Cholinergic synapse	21 (0.69%)	ko04725	Organismal Systems	Nervous system
**154**	Serotonergic synapse	21 (0.69%)	ko04726	Organismal Systems	Nervous system
**155**	Mitophagy - animal	21 (0.69%)	ko04137	Cellular Processes	Transport and catabolism
**156**	Prostate cancer	21 (0.69%)	ko05215	Human Diseases	Cancers: Specific types
**157**	PPAR signaling pathway	21 (0.69%)	ko03320	Organismal Systems	Endocrine system
**158**	Inflammatory mediator regulation of TRP channels	20 (0.65%)	ko04750	Organismal Systems	Sensory system
**159**	Apoptosis - fly	20 (0.65%)	ko04214	Cellular Processes	Cell growth and death
**160**	Vasopressin-regulated water reabsorption	20 (0.65%)	ko04962	Organismal Systems	Excretory system
**161**	Protein export	20 (0.65%)	ko03060	Genetic Information Processing	Folding, sorting and degradation
**162**	Glioma	20 (0.65%)	ko05214	Human Diseases	Cancers: Specific types
**163**	DNA replication	20 (0.65%)	ko03030	Genetic Information Processing	Replication and repair
**164**	EGFR tyrosine kinase inhibitor resistance	20 (0.65%)	ko01521	Human Diseases	Drug resistance: Antineoplastic
**165**	Acute myeloid leukemia	20 (0.65%)	ko05221	Human Diseases	Cancers: Specific types
**166**	Parathyroid hormone synthesis, secretion and action	20 (0.65%)	ko04928	Organismal Systems	Endocrine system
**167**	Pentose phosphate pathway	19 (0.62%)	ko00030	Metabolism	Carbohydrate metabolism
**168**	Longevity regulating pathway	19 (0.62%)	ko04211	Organismal Systems	Aging
**169**	Aldosterone synthesis and secretion	19 (0.62%)	ko04925	Organismal Systems	Endocrine system
**170**	Protein digestion and absorption	19 (0.62%)	ko04974	Organismal Systems	Digestive system

**171**	Choline metabolism in cancer	19 (0.62%)	ko05231	Human Diseases	Cancers: Overview
**172**	Cholesterol metabolism	19 (0.62%)	ko04979	Organismal Systems	Digestive system
**173**	B cell receptor signaling pathway	19 (0.62%)	ko04662	Organismal Systems	Immune system
**174**	Pancreatic secretion	19 (0.62%)	ko04972	Organismal Systems	Digestive system
**175**	Salivary secretion	19 (0.62%)	ko04970	Organismal Systems	Digestive system
**176**	Long-term depression	19 (0.62%)	ko04730	Organismal Systems	Nervous system
**177**	Natural killer cell mediated cytotoxicity	19 (0.62%)	ko04650	Organismal Systems	Immune system
**178**	Rheumatoid arthritis	19 (0.62%)	ko05323	Human Diseases	Immune diseases
**179**	Alanine, aspartate and glutamate metabolism	18 (0.59%)	ko00250	Metabolism	Amino acid metabolism
**180**	Gastric acid secretion	18 (0.59%)	ko04971	Organismal Systems	Digestive system
**181**	Breast cancer	18 (0.59%)	ko05224	Human Diseases	Cancers: Specific types
**182**	Endocrine resistance	18 (0.59%)	ko01522	Human Diseases	Drug resistance: Antineoplastic
**183**	Longevity regulating pathway - multiple species	18 (0.59%)	ko04213	Organismal Systems	Aging
**184**	Circadian entrainment	18 (0.59%)	ko04713	Organismal Systems	Environmental adaptation
**185**	Amyotrophic lateral sclerosis (ALS)	18 (0.59%)	ko05014	Human Diseases	Neurodegenerative diseases
**186**	Fructose and mannose metabolism	18 (0.59%)	ko00051	Metabolism	Carbohydrate metabolism
**187**	Phosphatidylinositol signaling system	18 (0.59%)	ko04070	Environmental Information Processing	Signal transduction
**188**	Glyoxylate and dicarboxylate metabolism	18 (0.59%)	ko00630	Metabolism	Carbohydrate metabolism
**189**	Melanogenesis	18 (0.59%)	ko04916	Organismal Systems	Endocrine system
**190**	VEGF signaling pathway	18 (0.59%)	ko04370	Environmental Information Processing	Signal transduction
**191**	Endometrial cancer	17 (0.56%)	ko05213	Human Diseases	Cancers: Specific types
**192**	Fc epsilon RI signaling pathway	17 (0.56%)	ko04664	Organismal Systems	Immune system
**193**	Nucleotide excision repair	17 (0.56%)	ko03420	Genetic Information Processing	Replication and repair
**194**	Thyroid cancer	17 (0.56%)	ko05216	Human Diseases	Cancers: Specific types
**195**	Prolactin signaling pathway	17 (0.56%)	ko04917	Organismal Systems	Endocrine system
**196**	Metabolism of xenobiotics by cytochrome P450	17 (0.56%)	ko00980	Metabolism	Xenobiotics biodegradation and metabolism
**197**	Tryptophan metabolism	16 (0.52%)	ko00380	Metabolism	Amino acid metabolism
**198**	Th17 cell differentiation	16 (0.52%)	ko04659	Organismal Systems	Immune system
**199**	Toll and Imd signaling pathway	16 (0.52%)	ko04624	Organismal Systems	Immune system
**200**	Signaling pathways regulating pluripotency of stem cells	16 (0.52%)	ko04550	Cellular Processes	Cellular community - eukaryotes
**201**	TGF-beta signaling pathway	16 (0.52%)	ko04350	Environmental Information Processing	Signal transduction
**202**	GABAergic synapse	16 (0.52%)	ko04727	Organismal Systems	Nervous system
**203**	Mismatch repair	16 (0.52%)	ko03430	Genetic Information Processing	Replication and repair

**204**	Inositol phosphate metabolism	16 (0.52%)	ko00562	Metabolism	Carbohydrate metabolism
**205**	beta-Alanine metabolism	16 (0.52%)	ko00410	Metabolism	Metabolism of other amino acids
**206**	Endocrine and other factor-regulated calcium reabsorption	16 (0.52%)	ko04961	Organismal Systems	Excretory system
**207**	Galactose metabolism	15 (0.49%)	ko00052	Metabolism	Carbohydrate metabolism
**208**	Leishmaniasis	15 (0.49%)	ko05140	Human Diseases	Infectious diseases: Parasitic
**209**	Non-small cell lung cancer	15 (0.49%)	ko05223	Human Diseases	Cancers: Specific types
**210**	Thyroid hormone synthesis	15 (0.49%)	ko04918	Organismal Systems	Endocrine system
**211**	Jak-STAT signaling pathway	15 (0.49%)	ko04630	Environmental Information Processing	Signal transduction
**212**	Mineral absorption	15 (0.49%)	ko04978	Organismal Systems	Digestive system
**213**	Chemical carcinogenesis	15 (0.49%)	ko05204	Human Diseases	Cancers: Overview
**214**	Glycerophospholipid metabolism	15 (0.49%)	ko00564	Metabolism	Lipid metabolism
**215**	Adipocytokine signaling pathway	14 (0.46%)	ko04920	Organismal Systems	Endocrine system
**216**	Phototransduction - fly	14 (0.46%)	ko04745	Organismal Systems	Sensory system
**217**	Butanoate metabolism	14 (0.46%)	ko00650	Metabolism	Carbohydrate metabolism
**218**	Melanoma	14 (0.46%)	ko05218	Human Diseases	Cancers: Specific types
**219**	Renin secretion	14 (0.46%)	ko04924	Organismal Systems	Endocrine system
**220**	Insulin secretion	13 (0.43%)	ko04911	Organismal Systems	Endocrine system
**221**	Porphyrin and chlorophyll metabolism	13 (0.43%)	ko00860	Metabolism	Metabolism of cofactors and vitamins
**222**	Amphetamine addiction	13 (0.43%)	ko05031	Human Diseases	Substance dependence
**223**	Fatty acid elongation	13 (0.43%)	ko00062	Metabolism	Lipid metabolism
**224**	SNARE interactions in vesicular transport	13 (0.43%)	ko04130	Genetic Information Processing	Folding, sorting and degradation
**225**	RIG-I-like receptor signaling pathway	13 (0.43%)	ko04622	Organismal Systems	Immune system
**226**	Bladder cancer	13 (0.43%)	ko05219	Human Diseases	Cancers: Specific types
**227**	Glycerolipid metabolism	12 (0.39%)	ko00561	Metabolism	Lipid metabolism
**228**	African trypanosomiasis	12 (0.39%)	ko05143	Human Diseases	Infectious diseases: Parasitic
**229**	Hematopoietic cell lineage	12 (0.39%)	ko04640	Organismal Systems	Immune system
**230**	Drug metabolism - cytochrome P450	12 (0.39%)	ko00982	Metabolism	Xenobiotics biodegradation and metabolism
**231**	Neuroactive ligand-receptor interaction	12 (0.39%)	ko04080	Environmental Information Processing	Signaling molecules and interaction
**232**	2-Oxocarboxylic acid metabolism	12 (0.39%)	ko01210	Metabolism	Global and overview maps
**233**	Tyrosine metabolism	12 (0.39%)	ko00350	Metabolism	Amino acid metabolism
**234**	Glycine, serine and threonine metabolism	12 (0.39%)	ko00260	Metabolism	Amino acid metabolism
**235**	Terpenoid backbone biosynthesis	11 (0.36%)	ko00900	Metabolism	Metabolism of terpenoids and polyketides

**236**	N-Glycan biosynthesis	11 (0.36%)	ko00510	Metabolism	Glycan biosynthesis and metabolism
**237**	Apoptosis - multiple species	11 (0.36%)	ko04215	Cellular Processes	Cell growth and death
**238**	Morphine addiction	11 (0.36%)	ko05032	Human Diseases	Substance dependence
**239**	Base excision repair	11 (0.36%)	ko03410	Genetic Information Processing	Replication and repair
**240**	Staphylococcus aureus infection	11 (0.36%)	ko05150	Human Diseases	Infectious diseases: Bacterial
**241**	Prion diseases	11 (0.36%)	ko05020	Human Diseases	Neurodegenerative diseases
**242**	Autophagy - other	11 (0.36%)	ko04136	Cellular Processes	Transport and catabolism
**243**	Collecting duct acid secretion	11 (0.36%)	ko04966	Organismal Systems	Excretory system
**244**	Cytosolic DNA-sensing pathway	11 (0.36%)	ko04623	Organismal Systems	Immune system
**245**	Olfactory transduction	10 (0.33%)	ko04740	Organismal Systems	Sensory system
**246**	Arachidonic acid metabolism	10 (0.33%)	ko00590	Metabolism	Lipid metabolism
**247**	Bile secretion	10 (0.33%)	ko04976	Organismal Systems	Digestive system
**248**	Ether lipid metabolism	10 (0.33%)	ko00565	Metabolism	Lipid metabolism
**249**	Biosynthesis of unsaturated fatty acids	10 (0.33%)	ko01040	Metabolism	Lipid metabolism
**250**	Th1 and Th2 cell differentiation	10 (0.33%)	ko04658	Organismal Systems	Immune system
**251**	Starch and sucrose metabolism	10 (0.33%)	ko00500	Metabolism	Carbohydrate metabolism
**252**	Steroid biosynthesis	9 (0.29%)	ko00100	Metabolism	Lipid metabolism
**253**	Aldosterone-regulated sodium reabsorption	9 (0.29%)	ko04960	Organismal Systems	Excretory system
**254**	Proximal tubule bicarbonate reclamation	9 (0.29%)	ko04964	Organismal Systems	Excretory system
**255**	Malaria	9 (0.29%)	ko05144	Human Diseases	Infectious diseases: Parasitic
**256**	Nicotinate and nicotinamide metabolism	9 (0.29%)	ko00760	Metabolism	Metabolism of cofactors and vitamins
**257**	Sphingolipid metabolism	9 (0.29%)	ko00600	Metabolism	Lipid metabolism
**258**	Cocaine addiction	9 (0.29%)	ko05030	Human Diseases	Substance dependence
**259**	Notch signaling pathway	8 (0.26%)	ko04330	Environmental Information Processing	Signal transduction
**260**	Type II diabetes mellitus	8 (0.26%)	ko04930	Human Diseases	Endocrine and metabolic diseases
**261**	One carbon pool by folate	8 (0.26%)	ko00670	Metabolism	Metabolism of cofactors and vitamins
**262**	Regulation of lipolysis in adipocytes	8 (0.26%)	ko04923	Organismal Systems	Endocrine system
**263**	Arginine biosynthesis	8 (0.26%)	ko00220	Metabolism	Amino acid metabolism
**264**	Pentose and glucuronate interconversions	8 (0.26%)	ko00040	Metabolism	Carbohydrate metabolism
**265**	Cytokine-cytokine receptor interaction	8 (0.26%)	ko04060	Environmental Information Processing	Signaling molecules and interaction
**266**	Histidine metabolism	8 (0.26%)	ko00340	Metabolism	Amino acid metabolism
**267**	Folate biosynthesis	8 (0.26%)	ko00790	Metabolism	Metabolism of cofactors and vitamins
**268**	Antifolate resistance	8 (0.26%)	ko01523	Human Diseases	Drug resistance: Antineoplastic

**269**	RNA polymerase	8 (0.26%)	ko03020	Genetic Information Processing	Transcription
**270**	Cortisol synthesis and secretion	7 (0.23%)	ko04927	Organismal Systems	Endocrine system
**271**	Dorso-ventral axis formation	7 (0.23%)	ko04320	Organismal Systems	Development
**272**	Inflammatory bowel disease (IBD)	7 (0.23%)	ko05321	Human Diseases	Immune diseases
**273**	Ascorbate and aldarate metabolism	7 (0.23%)	ko00053	Metabolism	Carbohydrate metabolism
**274**	Sulfur metabolism	7 (0.23%)	ko00920	Metabolism	Energy metabolism
**275**	Carbohydrate digestion and absorption	7 (0.23%)	ko04973	Organismal Systems	Digestive system
**276**	Hedgehog signaling pathway - fly	7 (0.23%)	ko04341	Environmental Information Processing	Signal transduction
**277**	Other glycan degradation	7 (0.23%)	ko00511	Metabolism	Glycan biosynthesis and metabolism
**278**	Glycosaminoglycan degradation	6 (0.2%)	ko00531	Metabolism	Glycan biosynthesis and metabolism
**279**	Other types of O-glycan biosynthesis	6 (0.2%)	ko00514	Metabolism	Glycan biosynthesis and metabolism
**280**	Synthesis and degradation of ketone bodies	6 (0.2%)	ko00072	Metabolism	Lipid metabolism
**281**	Phenylalanine metabolism	6 (0.2%)	ko00360	Metabolism	Amino acid metabolism
**282**	Fatty acid biosynthesis	6 (0.2%)	ko00061	Metabolism	Lipid metabolism
**283**	Allograft rejection	6 (0.2%)	ko05330	Human Diseases	Immune diseases
**284**	Hedgehog signaling pathway	6 (0.2%)	ko04340	Environmental Information Processing	Signal transduction
**285**	Renin-angiotensin system	6 (0.2%)	ko04614	Organismal Systems	Endocrine system
**286**	Selenocompound metabolism	6 (0.2%)	ko00450	Metabolism	Metabolism of other amino acids
**287**	Autoimmune thyroid disease	6 (0.2%)	ko05320	Human Diseases	Immune diseases
**288**	Type I diabetes mellitus	6 (0.2%)	ko04940	Human Diseases	Endocrine and metabolic diseases
**289**	Glycosphingolipid biosynthesis - globo and isoglobo series	6 (0.2%)	ko00603	Metabolism	Glycan biosynthesis and metabolism
**290**	Steroid hormone biosynthesis	5 (0.16%)	ko00140	Metabolism	Lipid metabolism
**291**	Thiamine metabolism	5 (0.16%)	ko00730	Metabolism	Metabolism of cofactors and vitamins
**292**	Hippo signaling pathway - multiple species	5 (0.16%)	ko04392	Environmental Information Processing	Signal transduction
**293**	Ovarian steroidogenesis	5 (0.16%)	ko04913	Organismal Systems	Endocrine system
**294**	Phototransduction	5 (0.16%)	ko04744	Organismal Systems	Sensory system
**295**	Homologous recombination	5 (0.16%)	ko03440	Genetic Information Processing	Replication and repair
**296**	Graft-versus-host disease	4 (0.13%)	ko05332	Human Diseases	Immune diseases
**297**	Glycosylphosphatidylinositol (GPI)-anchor biosynthesis	4 (0.13%)	ko00563	Metabolism	Glycan biosynthesis and metabolism
**298**	Pantothenate and CoA biosynthesis	4 (0.13%)	ko00770	Metabolism	Metabolism of cofactors and vitamins
**299**	Taste transduction	4 (0.13%)	ko04742	Organismal Systems	Sensory system
**300**	Basal transcription factors	4 (0.13%)	ko03022	Genetic Information Processing	Transcription
**301**	Riboflavin metabolism	4 (0.13%)	ko00740	Metabolism	Metabolism of cofactors and

					vitamins
**302**	Glycosphingolipid biosynthesis - ganglio series	4 (0.13%)	ko00604	Metabolism	Glycan biosynthesis and metabolism
**303**	alpha-Linolenic acid metabolism	4 (0.13%)	ko00592	Metabolism	Lipid metabolism
**304**	Circadian rhythm	3 (0.1%)	ko04710	Organismal Systems	Environmental adaptation
**305**	Fanconi anemia pathway	3 (0.1%)	ko03460	Genetic Information Processing	Replication and repair
**306**	Mucin type O-glycan biosynthesis	3 (0.1%)	ko00512	Metabolism	Glycan biosynthesis and metabolism
**307**	Primary bile acid biosynthesis	3 (0.1%)	ko00120	Metabolism	Lipid metabolism
**308**	Primary immunodeficiency	3 (0.1%)	ko05340	Human Diseases	Immune diseases
**309**	Phosphonate and phosphinate metabolism	3 (0.1%)	ko00440	Metabolism	Metabolism of other amino acids
**310**	Insect hormone biosynthesis	3 (0.1%)	ko00981	Metabolism	Metabolism of terpenoids and polyketides
**311**	Fat digestion and absorption	3 (0.1%)	ko04975	Organismal Systems	Digestive system
**312**	Vitamin B6 metabolism	3 (0.1%)	ko00750	Metabolism	Metabolism of cofactors and vitamins
**313**	Basal cell carcinoma	3 (0.1%)	ko05217	Human Diseases	Cancers: Specific types
**314**	Retinol metabolism	3 (0.1%)	ko00830	Metabolism	Metabolism of cofactors and vitamins
**315**	Sulfur relay system	3 (0.1%)	ko04122	Genetic Information Processing	Folding, sorting and degradation
**316**	Vitamin digestion and absorption	3 (0.1%)	ko04977	Organismal Systems	Digestive system
**317**	Nitrogen metabolism	3 (0.1%)	ko00910	Metabolism	Energy metabolism
**318**	Ubiquinone and other terpenoid-quinone biosynthesis	2 (0.07%)	ko00130	Metabolism	Metabolism of cofactors and vitamins
**319**	D-Glutamine and D-glutamate metabolism	2 (0.07%)	ko00471	Metabolism	Metabolism of other amino acids
**320**	Phenylalanine, tyrosine and tryptophan biosynthesis	2 (0.07%)	ko00400	Metabolism	Amino acid metabolism
**321**	Asthma	2 (0.07%)	ko05310	Human Diseases	Immune diseases
**322**	Neomycin, kanamycin and gentamicin biosynthesis	2 (0.07%)	ko00524	Metabolism	Biosynthesis of other secondary metabolites
**323**	Mannose type O-glycan biosynthesis	2 (0.07%)	ko00515	Metabolism	Glycan biosynthesis and metabolism
**324**	Non-homologous end-joining	2 (0.07%)	ko03450	Genetic Information Processing	Replication and repair
**325**	Biotin metabolism	2 (0.07%)	ko00780	Metabolism	Metabolism of cofactors and vitamins
**326**	Nicotine addiction	2 (0.07%)	ko05033	Human Diseases	Substance dependence
**327**	Intestinal immune network for IgA production	2 (0.07%)	ko04672	Organismal Systems	Immune system
**328**	Cutin, suberine and wax biosynthesis	2 (0.07%)	ko00073	Metabolism	Lipid metabolism

**329**	Caffeine metabolism	1 (0.03%)	ko00232	Metabolism	Biosynthesis of other secondary metabolites
**330**	Valine, leucine and isoleucine biosynthesis	1 (0.03%)	ko00290	Metabolism	Amino acid metabolism
**331**	Linoleic acid metabolism	1 (0.03%)	ko00591	Metabolism	Lipid metabolism
**332**	Glycosaminoglycan biosynthesis - heparan sulfate / heparin	1 (0.03%)	ko00534	Metabolism	Glycan biosynthesis and metabolism
**333**	Taurine and hypotaurine metabolism	1 (0.03%)	ko00430	Metabolism	Metabolism of other amino acids
**334**	Glycosaminoglycan biosynthesis - chondroitin sulfate / dermatan sulfate	1 (0.03%)	ko00532	Metabolism	Glycan biosynthesis and metabolism
**335**	Monobactam biosynthesis	1 (0.03%)	ko00261	Metabolism	Biosynthesis of other secondary metabolites
**336**	Circadian rhythm - fly	1 (0.03%)	ko04711	Organismal Systems	Environmental adaptation
**337**	ABC transporters	1 (0.03%)	ko02010	Environmental Information Processing	Membrane transport

Table S2. Identified DEPs in *IRS1* silenced GMECs

Primary_protein_ID	ratio	*P* value	Class	Description
tr|A0A8C2P961|A0A8C2P961_CAPHI	1.679766	0.00638	Up	Uncharacterized protein OS=Capra hircus OX=9925 PE=4 SV=1
tr|A0A452GAM5|A0A452GAM5_CAPHI	80.58179	0.007151	Up	Keratin 82 OS=Capra hircus OX=9925 GN=KRT82 PE=3 SV=1
tr|A0A452EY08|A0A452EY08_CAPHI	0.651776	0.018808	Down	Protein arginine N-methyltransferase 5 OS=Capra hircus OX=9925 GN=PRMT5 PE=3 SV=1
tr|A0A8C2S8I8|A0A8C2S8I8_CAPHI	6.440772	0.019999	Up	MAP7 domain containing 1 OS=Capra hircus OX=9925 PE=4 SV=1
tr|A0A452ENI2|A0A452ENI2_CAPHI	1.794928	0.021819	Up	Syntaxin 7 OS=Capra hircus OX=9925 GN=STX7 PE=3 SV=1
tr|A0A452FPA3|A0A452FPA3_CAPHI	2.205146	0.025801	Up	KIAA1217 ortholog OS=Capra hircus OX=9925 GN=KIAA1217 PE=4 SV=1
tr|A0A8C2SJN8|A0A8C2SJN8_CAPHI	1.598218	0.027044	Up	Phosphodiesterase 12 OS=Capra hircus OX=9925 GN=PDE12 PE=4 SV=1
tr|A0A8C2RAM0|A0A8C2RAM0_CAPHI	0.431634	0.030581	Down	Glutaredoxin 5 OS=Capra hircus OX=9925 PE=4 SV=1
tr|A0A452EKV1|A0A452EKV1_CAPHI	0.584772	0.033474	Down	Protein MAK16 homolog OS=Capra hircus OX=9925 PE=3 SV=1
tr|A0A452FS44|A0A452FS44_CAPHI	2.275074	0.038024	Up	Heme binding protein 2 OS=Capra hircus OX=9925 GN=HEBP2 PE=3 SV=1
tr|A0A452E2T3|A0A452E2T3_CAPHI	5.679159	0.043977	Up	Rhophilin Rho GTPase binding protein 2 OS=Capra hircus OX=9925 GN=RHPN2 PE=4 SV=1
tr|A0A8C2SL02|A0A8C2SL02_CAPHI	1.63364	0.04659	Up	Myosin XVIIIA OS=Capra hircus OX=9925 PE=4 SV=1

Table S3. GO enriched DEPs

Ontoloty	GO_term	Protein_ID	log_2Foldchange	Up/Down
biological_process	biological regulation	tr|A0A452E2T3|A0A452E2T3_CAPHI	2.505677	up
biological_process	biological regulation	tr|A0A452ENI2|A0A452ENI2_CAPHI	0.843926	up
biological_process	biological regulation	tr|A0A452EY08|A0A452EY08_CAPHI	-0.61755	down
biological_process	biological regulation	tr|A0A452FS44|A0A452FS44_CAPHI	1.185913	up
biological_process	biological regulation	tr|A0A8C2P961|A0A8C2P961_CAPHI	0.74826	up
biological_process	biological regulation	tr|A0A8C2RAM0|A0A8C2RAM0_CAPHI	-1.21212	down
biological_process	biological regulation	tr|A0A8C2SJN8|A0A8C2SJN8_CAPHI	0.676464	up
biological_process	cell killing	tr|A0A452ENI2|A0A452ENI2_CAPHI	0.843926	up
biological_process	cellular component organization or biogenesis	tr|A0A452EKV1|A0A452EKV1_CAPHI	-0.77405	down
biological_process	cellular component organization or biogenesis	tr|A0A452ENI2|A0A452ENI2_CAPHI	0.843926	up
biological_process	cellular component organization or biogenesis	tr|A0A452EY08|A0A452EY08_CAPHI	-0.61755	down
biological_process	cellular component organization or biogenesis	tr|A0A452FS44|A0A452FS44_CAPHI	1.185913	up
biological_process	cellular process	tr|A0A452E2T3|A0A452E2T3_CAPHI	2.505677	up
biological_process	cellular process	tr|A0A452EKV1|A0A452EKV1_CAPHI	-0.77405	down
biological_process	cellular process	tr|A0A452ENI2|A0A452ENI2_CAPHI	0.843926	up
biological_process	cellular process	tr|A0A452EY08|A0A452EY08_CAPHI	-0.61755	down
biological_process	cellular process	tr|A0A452FS44|A0A452FS44_CAPHI	1.185913	up
biological_process	cellular process	tr|A0A8C2P961|A0A8C2P961_CAPHI	0.74826	up
biological_process	cellular process	tr|A0A8C2RAM0|A0A8C2RAM0_CAPHI	-1.21212	down
biological_process	cellular process	tr|A0A8C2SJN8|A0A8C2SJN8_CAPHI	0.676464	up
biological_process	developmental process	tr|A0A452FPA3|A0A452FPA3_CAPHI	1.140874	up
biological_process	developmental process	tr|A0A8C2P961|A0A8C2P961_CAPHI	0.74826	up
biological_process	immune system process	tr|A0A452ENI2|A0A452ENI2_CAPHI	0.843926	up
biological_process	immune system process	tr|A0A8C2SJN8|A0A8C2SJN8_CAPHI	0.676464	up

biological_process	localization	tr|A0A452ENI2|A0A452ENI2_CAPHI	0.843926	up
biological_process	localization	tr|A0A452FS44|A0A452FS44_CAPHI	1.185913	up
biological_process	metabolic process	tr|A0A452EKV1|A0A452EKV1_CAPHI	-0.77405	down
biological_process	metabolic process	tr|A0A452EY08|A0A452EY08_CAPHI	-0.61755	down
biological_process	metabolic process	tr|A0A8C2P961|A0A8C2P961_CAPHI	0.74826	up
biological_process	metabolic process	tr|A0A8C2RAM0|A0A8C2RAM0_CAPHI	-1.21212	down
biological_process	metabolic process	tr|A0A8C2SJN8|A0A8C2SJN8_CAPHI	0.676464	up
biological_process	multi-organism process	tr|A0A8C2SJN8|A0A8C2SJN8_CAPHI	0.676464	up
biological_process	multicellular organismal process	tr|A0A452EY08|A0A452EY08_CAPHI	-0.61755	down
biological_process	multicellular organismal process	tr|A0A452FPA3|A0A452FPA3_CAPHI	1.140874	up
biological_process	negative regulation of biological process	tr|A0A8C2SJN8|A0A8C2SJN8_CAPHI	0.676464	up
biological_process	positive regulation of biological process	tr|A0A452ENI2|A0A452ENI2_CAPHI	0.843926	up
biological_process	positive regulation of biological process	tr|A0A452EY08|A0A452EY08_CAPHI	-0.61755	down
biological_process	positive regulation of biological process	tr|A0A452FS44|A0A452FS44_CAPHI	1.185913	up
biological_process	positive regulation of biological process	tr|A0A8C2P961|A0A8C2P961_CAPHI	0.74826	up
biological_process	positive regulation of biological process	tr|A0A8C2SJN8|A0A8C2SJN8_CAPHI	0.676464	up
biological_process	regulation of biological process	tr|A0A452E2T3|A0A452E2T3_CAPHI	2.505677	up
biological_process	regulation of biological process	tr|A0A452ENI2|A0A452ENI2_CAPHI	0.843926	up
biological_process	regulation of biological process	tr|A0A452EY08|A0A452EY08_CAPHI	-0.61755	down
biological_process	regulation of biological process	tr|A0A452FS44|A0A452FS44_CAPHI	1.185913	up
biological_process	regulation of biological process	tr|A0A8C2P961|A0A8C2P961_CAPHI	0.74826	up
biological_process	regulation of biological process	tr|A0A8C2RAM0|A0A8C2RAM0_CAPHI	-1.21212	down
biological_process	regulation of biological process	tr|A0A8C2SJN8|A0A8C2SJN8_CAPHI	0.676464	up
biological_process	response to stimulus	tr|A0A452E2T3|A0A452E2T3_CAPHI	2.505677	up
biological_process	response to stimulus	tr|A0A452ENI2|A0A452ENI2_CAPHI	0.843926	up
biological_process	response to stimulus	tr|A0A452EY08|A0A452EY08_CAPHI	-0.61755	down

biological_process	response to stimulus	tr|A0A8C2P961|A0A8C2P961_CAPHI	0.74826	up
biological_process	response to stimulus	tr|A0A8C2SJN8|A0A8C2SJN8_CAPHI	0.676464	up
biological_process	rhythmic process	tr|A0A452EY08|A0A452EY08_CAPHI	-0.61755	down
biological_process	signaling	tr|A0A452E2T3|A0A452E2T3_CAPHI	2.505677	up
biological_process	signaling	tr|A0A452EY08|A0A452EY08_CAPHI	-0.61755	down
cellular_component	cell	tr|A0A452E2T3|A0A452E2T3_CAPHI	2.505677	up
cellular_component	cell	tr|A0A452EKV1|A0A452EKV1_CAPHI	-0.77405	down
cellular_component	cell	tr|A0A452ENI2|A0A452ENI2_CAPHI	0.843926	up
cellular_component	cell	tr|A0A452EY08|A0A452EY08_CAPHI	-0.61755	down
cellular_component	cell	tr|A0A452FS44|A0A452FS44_CAPHI	1.185913	up
cellular_component	cell	tr|A0A452GAM5|A0A452GAM5_CAPHI	6.332382	up
cellular_component	cell	tr|A0A8C2P961|A0A8C2P961_CAPHI	0.74826	up
cellular_component	cell	tr|A0A8C2RAM0|A0A8C2RAM0_CAPHI	-1.21212	down
cellular_component	cell	tr|A0A8C2SJN8|A0A8C2SJN8_CAPHI	0.676464	up
cellular_component	cell part	tr|A0A452E2T3|A0A452E2T3_CAPHI	2.505677	up
cellular_component	cell part	tr|A0A452EKV1|A0A452EKV1_CAPHI	-0.77405	down
cellular_component	cell part	tr|A0A452ENI2|A0A452ENI2_CAPHI	0.843926	up
cellular_component	cell part	tr|A0A452EY08|A0A452EY08_CAPHI	-0.61755	down
cellular_component	cell part	tr|A0A452FS44|A0A452FS44_CAPHI	1.185913	up
cellular_component	cell part	tr|A0A452GAM5|A0A452GAM5_CAPHI	6.332382	up
cellular_component	cell part	tr|A0A8C2P961|A0A8C2P961_CAPHI	0.74826	up
cellular_component	cell part	tr|A0A8C2RAM0|A0A8C2RAM0_CAPHI	-1.21212	down
cellular_component	cell part	tr|A0A8C2SJN8|A0A8C2SJN8_CAPHI	0.676464	up
cellular_component	extracellular region	tr|A0A452ENI2|A0A452ENI2_CAPHI	0.843926	up
cellular_component	extracellular region	tr|A0A452FS44|A0A452FS44_CAPHI	1.185913	up
cellular_component	extracellular region	tr|A0A8C2P961|A0A8C2P961_CAPHI	0.74826	up

cellular_component	extracellular region part	tr|A0A452ENI2|A0A452ENI2_CAPHI	0.843926	up
cellular_component	extracellular region part	tr|A0A452FS44|A0A452FS44_CAPHI	1.185913	up
cellular_component	extracellular region part	tr|A0A8C2P961|A0A8C2P961_CAPHI	0.74826	up
cellular_component	macromolecular complex	tr|A0A452EKV1|A0A452EKV1_CAPHI	-0.77405	down
cellular_component	macromolecular complex	tr|A0A452ENI2|A0A452ENI2_CAPHI	0.843926	up
cellular_component	macromolecular complex	tr|A0A452EY08|A0A452EY08_CAPHI	-0.61755	down
cellular_component	membrane	tr|A0A452ENI2|A0A452ENI2_CAPHI	0.843926	up
cellular_component	membrane part	tr|A0A452ENI2|A0A452ENI2_CAPHI	0.843926	up
cellular_component	membrane-enclosed lumen	tr|A0A452EKV1|A0A452EKV1_CAPHI	-0.77405	down
cellular_component	membrane-enclosed lumen	tr|A0A452EY08|A0A452EY08_CAPHI	-0.61755	down
cellular_component	membrane-enclosed lumen	tr|A0A8C2P961|A0A8C2P961_CAPHI	0.74826	up
cellular_component	membrane-enclosed lumen	tr|A0A8C2RAM0|A0A8C2RAM0_CAPHI	-1.21212	down
cellular_component	membrane-enclosed lumen	tr|A0A8C2SJN8|A0A8C2SJN8_CAPHI	0.676464	up
cellular_component	organelle	tr|A0A452EKV1|A0A452EKV1_CAPHI	-0.77405	down
cellular_component	organelle	tr|A0A452ENI2|A0A452ENI2_CAPHI	0.843926	up
cellular_component	organelle	tr|A0A452EY08|A0A452EY08_CAPHI	-0.61755	down
cellular_component	organelle	tr|A0A452FS44|A0A452FS44_CAPHI	1.185913	up
cellular_component	organelle	tr|A0A452GAM5|A0A452GAM5_CAPHI	6.332382	up
cellular_component	organelle	tr|A0A8C2P961|A0A8C2P961_CAPHI	0.74826	up
cellular_component	organelle	tr|A0A8C2RAM0|A0A8C2RAM0_CAPHI	-1.21212	down
cellular_component	organelle	tr|A0A8C2SJN8|A0A8C2SJN8_CAPHI	0.676464	up
cellular_component	organelle part	tr|A0A452EKV1|A0A452EKV1_CAPHI	-0.77405	down
cellular_component	organelle part	tr|A0A452ENI2|A0A452ENI2_CAPHI	0.843926	up
cellular_component	organelle part	tr|A0A452EY08|A0A452EY08_CAPHI	-0.61755	down
cellular_component	organelle part	tr|A0A452GAM5|A0A452GAM5_CAPHI	6.332382	up
cellular_component	organelle part	tr|A0A8C2P961|A0A8C2P961_CAPHI	0.74826	up

cellular_component	organelle part	tr|A0A8C2RAM0|A0A8C2RAM0_CAPHI	-1.21212	down
cellular_component	organelle part	tr|A0A8C2SJN8|A0A8C2SJN8_CAPHI	0.676464	up
cellular_component	supramolecular complex	tr|A0A452GAM5|A0A452GAM5_CAPHI	6.332382	up
molecular_function	binding	tr|A0A452EKV1|A0A452EKV1_CAPHI	-0.77405	down
molecular_function	binding	tr|A0A452ENI2|A0A452ENI2_CAPHI	0.843926	up
molecular_function	binding	tr|A0A452EY08|A0A452EY08_CAPHI	-0.61755	down
molecular_function	binding	tr|A0A8C2RAM0|A0A8C2RAM0_CAPHI	-1.21212	down
molecular_function	binding	tr|A0A8C2SJN8|A0A8C2SJN8_CAPHI	0.676464	up
molecular_function	catalytic activity	tr|A0A452EY08|A0A452EY08_CAPHI	-0.61755	down
molecular_function	catalytic activity	tr|A0A8C2RAM0|A0A8C2RAM0_CAPHI	-1.21212	down
molecular_function	catalytic activity	tr|A0A8C2SJN8|A0A8C2SJN8_CAPHI	0.676464	up
molecular_function	molecular function regulator	tr|A0A452ENI2|A0A452ENI2_CAPHI	0.843926	up
molecular_function	structural molecule activity	tr|A0A452GAM5|A0A452GAM5_CAPHI	6.332382	up
molecular_function	transcription regulator activity	tr|A0A452EY08|A0A452EY08_CAPHI	-0.61755	down

Table S4. Subcellular Localization of DEPs

SeqID	Subcellular_location	Description
tr|A0A8C2P961|A0A8C2P961_CAPHI	cyto	Uncharacterized protein OS=Capra hircus OX=9925 PE=4 SV=1
tr|A0A452GAM5|A0A452GAM5_CAPHI	mito	Keratin 82 OS=Capra hircus OX=9925 GN=KRT82 PE=3 SV=1
tr|A0A452EY08|A0A452EY08_CAPHI	cyto_nucl	Protein arginine N-methyltransferase 5 OS=Capra hircus OX=9925 GN=PRMT5 PE=3 SV=1
tr|A0A8C2S8I8|A0A8C2S8I8_CAPHI	E.R.	MAP7 domain containing 1 OS=Capra hircus OX=9925 PE=4 SV=1
tr|A0A452ENI2|A0A452ENI2_CAPHI	plas	Syntaxin 7 OS=Capra hircus OX=9925 GN=STX7 PE=3 SV=1
tr|A0A452FPA3|A0A452FPA3_CAPHI	nucl	KIAA1217 ortholog OS=Capra hircus OX=9925 GN=KIAA1217 PE=4 SV=1
tr|A0A8C2SJN8|A0A8C2SJN8_CAPHI	mito	Phosphodiesterase 12 OS=Capra hircus OX=9925 GN=PDE12 PE=4 SV=1
tr|A0A8C2RAM0|A0A8C2RAM0_CAPHI	cyto	Glutaredoxin 5 OS=Capra hircus OX=9925 PE=4 SV=1
tr|A0A452EKV1|A0A452EKV1_CAPHI	cyto	Protein MAK16 homolog OS=Capra hircus OX=9925 PE=3 SV=1
tr|A0A452FS44|A0A452FS44_CAPHI	cyto	Heme binding protein 2 OS=Capra hircus OX=9925 GN=HEBP2 PE=3 SV=1
tr|A0A452E2T3|A0A452E2T3_CAPHI	nucl	Rhophilin Rho GTPase binding protein 2 OS=Capra hircus OX=9925 GN=RHPN2 PE=4 SV=1
tr|A0A8C2SL02|A0A8C2SL02_CAPHI	nucl	Myosin XVIIIA OS=Capra hircus OX=9925 PE=4 SV=1
